# Supplementary material for: Identification of photoperiod-induced specific miRNAs in the adrenal glands of Sunite sheep (Ovis aries)
Source: Front Vet Sci. 2022 Jul 22;9:888207. doi: 10.3389/fvets.2022.888207 (PMC9354845; doi:10.3389/fvets.2022.888207)
Supplement: Supplementary file 1 [file Data_Sheet_1.ZIP › Supplementary file/miRDeep2 file.zip!/miRDeep2 file/Novel_Structure/SP42A3/Novel_343.pdf]

Provisional ID	:	NC_019469.2_19196
Score total	:	1.1
Score for star read(s)	:	-1.3
Score for read counts	:	0
Score for mfe	:	0.8
Score for randfold	:	1.6
Score for cons. seed	:	
Total read count	:	20
Mature read count	:	20
Loop read count	:	0
Star read count	:	0

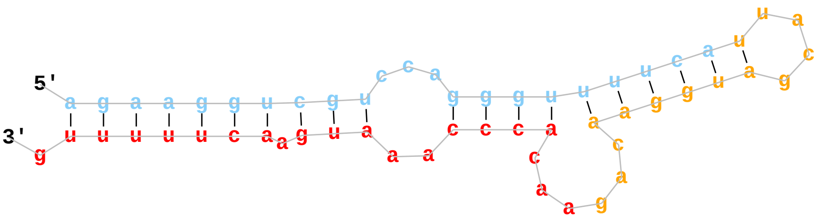

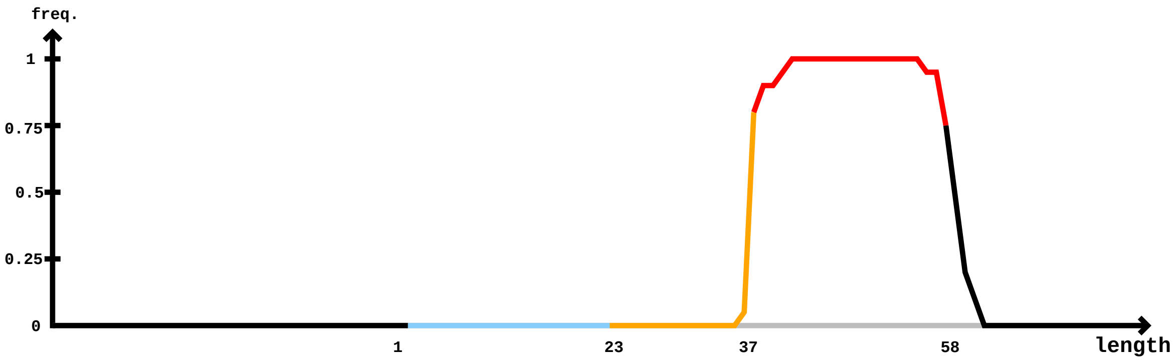


```
                                          Star                                      Mature
5'- agccacuggaacccccucuguuaucggguuggccaagaaggucguccaggguuuucauuacgauggaacagaacacccaaaugaacuuuuuggcaggcccaauaccaaucac   -3'    exp
    .......((.....))..((.(((.(((((.(((((((((.((((...((((((((((....))))))......))))..)))).))))))))).))))).))).)).....    reads     mm        sample
    ......................................................................gaacacccaaaugaacuuuuug....................    1         0         seq
    .......................................................................aaAacccaaaugaacuuu.......................    1         1         seq
    .......................................................................aaAacccaaaugaacuuuuu.....................    4         1         seq
    .......................................................................aaAacccaaaugaacuuuuug....................    9         1         seq
    .......................................................................aaGacccaaaugaacuuuuugg...................    1         1         seq
    ........................................................................aAacccaaaugaacuuuuuggc..................    2         1         seq
    ..........................................................................acccGaaugaacuuuuuggca.................    1         1         seq
    ...........................................................................cccGaaugaacuuuuuggca.................    1         1         seq
```
